# Naoluo Xintong Decoction Ameliorates Cerebral Ischemia-Reperfusion Injury by Promoting Angiogenesis through Activating the HIF-1*α*/VEGF Signaling Pathway in Rats

**DOI:** 10.1155/2022/9341466

**Published:** 2022-04-11

**Authors:** Pei-Pei Li, Ling He, Li-Miao Zhang, Xue-Mei Qin, Jian-Peng Hu

**Affiliations:** ^1^Key Laboratory of Xin'an Medicine, Ministry of Education, Anhui University of Chinese Medicine, Hefei, Anhui 230038, China; ^2^School of Chinese Medicine, Anhui University of Chinese Medicine, Hefei, Anhui 230012, China

## Abstract

**Background:**

Naoluo Xintong decoction (NLXTD) is a traditional Chinese medicine (TCM) formula which has been used to improve neuronal functional recovery after cerebral ischemic stroke. However, the molecular mechanism underlying NLXTD's amelioration of ischemic stroke remains unclear. The present study was designed to explore the effect and mechanism of NLXTD on brain angiogenesis in a rat model with cerebral ischemia-reperfusion (I/R) injury targeting the hypoxia-inducible factor-1*α* (HIF-1*α*)/vascular endothelial growth factor (VEGF) pathway.

**Materials and Methods:**

Cerebral I/R model was established by the classical middle cerebral artery occlusion (MCAO) method. Sprague-Dawley (SD) male rats (*n* = 80) were randomly divided into the sham-operation group, the model group, the HIF-1*α* inhibitor 2-methoxyestradiol (2ME2) group, the 2ME2 with NLXTD group, and the NLXTD group. Neurological deficit test, TTC staining, H&E staining, TUNEL staining, immunohistochemistry (IH), immunofluorescence (IF), western blot, and quantitative RT-PCR were performed to evaluate the effect of NLXTD after MCAO.

**Results:**

Administration of NLXTD significantly decreased neuron deficiency scores, reduced brain infarct volume, and lowered damaged and apoptotic cells after brain I/R injury in rats. Meanwhile, NLXTD had a protective effect on angiogenesis by increasing the MVD and the expressions of BrdU and CD34, which enhanced the number of endothelial cells in the ischemic penumbra brain. NLXTD treatment significantly raised the protein and mRNA levels of HIF-1*α*, VEGF, VEGFR2, and Notch1 compared with the model treatment. In contrast, a specific HIF-1*α* inhibitor, 2ME2, inhibited the improvement of neurological function and angiogenesis in NLXTD-induced rats with cerebral I/R injury, suggesting that NLXTD played a positive role in ischemic brain injury by activating the HIF-1*α*/VEGF signaling pathway.

**Conclusions:**

NLXTD exerts neuroprotection targeting angiogenesis by upregulating the HIF-1*α*/VEGF signaling pathway on cerebral I/R injury rats.

## 1. Introduction

Ischemic stroke is a cerebral vascular disease caused by a sudden obstruction of a blood vessel and the loss of blood supply to related brain areas [1], which is one of the leading causes of death and physical disability around the world [2]. Barreto reported that the global prevalence of ischemic stroke increased by 2.7% from 2006 to 2016 [3]. In the treatment of ischemic stroke, restoration of the blood supply can reduce the wider brain tissue injured by rescuing a reversible damaged penumbra of tissue [4]. Clinically, injection of recombinant tissue plasminogen activator (rtPA) is considered the most effective therapy for curing cerebral ischemia [5]. However, this therapy can lead to blood perfusion of brain tissue and may also cause serious complications, such as cerebral ischemia-reperfusion (I/R) injury during treatment [6]. Cerebral I/R injury is a deterioration of ischemic but repairable brain tissue after reperfusion [7] and is considered as the main culprit in the treatment of ischemic stroke [8]. Therefore, therapeutic options or novel drugs to reduce or minimize cerebral reperfusion injury are urgently needed [9].

Cerebrovascular generation is a process of repair and regeneration in the recovery of the body's neurological function after cerebral ischemia, and angiogenesis is the main form of cerebrovascular generation [10]. Studies have shown that angiogenesis is an important process of neurovascular unit remodeling after ischemic stroke [11], which not only increases oxygen and nutrition supply to the ischemic penumbra [12] but also modulates the growth of axons and neurogenesis, including proliferation, migration, and maturation of neural stem cells (NSCs) [13]. In addition, previous research has confirmed that the increase of microvessel density (MVD) in the peri-infarct area can contribute to a longer survival time in stroke patients, suggesting that the poststroke angiogenic response is important for neurological recovery [14]. Angiogenesis can protect the brain from damage during cerebral I/R, which may be a therapeutic strategy to improve poststroke functional recovery [15].

Multitarget therapy with herbal drugs has been considered as an alternative and complementary method for the treatment of ischemic stroke. Traditional Chinese medicine (TCM) has been applied in treating cerebral ischemia in China for thousands of years [16]. In the long history of TCM, various regional medical schools have been derived from different geographical environments, of which Xin'an Medicine School is a representative medical genre. The Xin'an Medical School is originated from the Jin Dynasty, developed in the Song Dynasty, flourished in the Ming and Qing Dynasties, and has been passed down to the modern era [17]. As an important school of Chinese medicine, Xin'an Medical School has rich and profound clinical experiences for the treatment and prevention of cardiocerebral vascular diseases, especially ischemic stroke. Naoluo Xintong decoction (NLXTD) is a classical Chinese herbal medicine formulation of Xin'an medicine school, which was created by Xin'an physician Wang Letao. Due to the fact that NLXTD is an empirical and effective prescription for ischemic stroke, it has been widely used in clinical practice [18].

NLXTD includes the 7 following kinds of herbs (Table 1) as recorded in the Chinese Pharmacopoeia: (1) *Radix Astragali*, (2) *Rhizoma Chuanxiong*, (3) *Radix et Rhizoma Notoginseng*, (4) *Rhizoma Gastrodiae*, (5) *Scolopendra*, (6) *Radix Angelicae Sinensis*, and (7) *Flos Carthami*, in the mass ratio of 15 : 5 : 3 : 5 : 2 : 5 : 5, respectively. Three-dimensional ultrafast high-performance liquid chromatography coupled with diode array detector (3D-UPLC-DAD) was used to verify the components of NLXTD in our previous research [19]. According to the TCM theory, NLXTD was used to reinforce energy (qi) flow through energy meridians and promote blood circulation to remove blood stasis. Our previous work has demonstrated that NLXTD could reduce cerebral I/R injury and improve neurological deficit by increasing blood supply to brain ischemic penumbra tissue, improving blood circulation and downregulating expression of genes involved in apoptosis, inflammation, and blood coagulation [20, 21]. A clinical research from Li et al. [22] found that the NLXTD combined with chemical drugs showed better effect than chemical drugs applied alone. In the group treated with NLXTD combined with chemical drugs, the National Institute of Health Stroke Scale (NIHSS) was lower than that in the group treated with chemical drugs, and the Barthel index (BI) was significantly higher than that in the chemical drugs group. Another experimental study showed that NLXTD played a protective role in I/R injury model rats by regulating vascular endothelial growth factor (VEGF) and promoting the formation of new blood vessels [23]. However, no studies have been performed to elucidate the brain protection mechanism of angiogenesis in NLXTD treatment.

Hypoxia-inducible factor-1*α* (HIF-1*α*) is a member of the hypoxia-inducible factor family, which is an important transcriptional regulator that stabilizes the intracellular environment during hypoxia and ischemia [24]. Studies have confirmed that HIF-1*α* can activate erythropoietin (EPO), glucose transporter-1/3 (GLU1/3), glycolytic enzymes, and other downstream target genes through transcription to participate in angiogenesis, erythropoiesis, energy metabolism, apoptosis, and other processes, to regulate the acute and chronic adaptation of brain tissue to hypoxia [25, 26]. Baranova [27] reported that neuron-specific knockout of the HIF-1 gene in mice can aggravate brain damage after MCAO and reduce the survival rate of mice. Zhou et al. [28] found that HIF-1*α* inhibitor 2-methoxyestradiol (2ME2) significantly reduced the HIF-1 levels and decreased neuronal survival in rats with global cerebral ischemia. While HIF-1*α* was found to be an essential participator in cerebral ischemia [29, 30], its potential role and its target genes in tissue injury after cerebral ischemia remain controversial [31]. Angiogenesis is a vital process of vascular reconstruction after cerebral ischemia. VEGF is a key factor of angiogenesis and growth, and it plays an important role in promoting the proliferation, differentiation, and migration of vascular endothelial cells [32]. It has been reported that the expression of VEGF is elevated in the ischemic penumbra within 3 hours following the ischemia. Previous studies have shown that HIF-1*α* mediates angiogenesis after cerebral ischemia and rescues ischemic penumbra of brain tissue by promoting the expression of its target gene, VEGF [33]. In addition, as a downstream product of HIF-1*α*, VEGF can form new microvessels and activate the Notch signaling pathway [34]. Li et al. [35] also found that HIF-1*α* can regulate the VEGF/Notch1 signaling pathway in the development of collateral circulation. In our previous experiments, we have reported that NLXTD can enhance VEFG expression in ischemic brain tissue and promote brain microvascular. However, the mechanism of NLXTD in promoting angiogenesis after cerebral ischemia and its relationship with HIF-1a, VEGF, and Notch is still unclear.

In brief, the purpose of this study was to determine whether a molecular cascade involving HIF-1*α*, VEGF, and Notch is causally related to the regulation of angiogenesis in brain tissues by treating a rat model of cerebral ischemia with NLXTD. Furthermore, we aimed to explore the main regulatory mechanism of the HIF-1a/VEGF pathway on angiogenesis in NLXTD and provide a new idea for the treatment of ischemic stroke.

## 2. Material and Methods

### 2.1. Ethical Statement

This study was performed according to protocols approved by the local animal ethic committee of the Anhui University of Chinese Medicine. All procedures involving animals and their care were performed in strict accordance with the Guidance for the Care and Use of Laboratory Animals issued by the US National Institute of Health (NIH publication no. 85-23, revised 1996).

### 2.2. Experimental Animals

Healthy male Sprague-Dawley (SD) rats (*n* = 80, *m* = 280 ± 20 g) were purchased from Anhui Medical University Laboratory Animal Center (license number: SCXK (Wan), 2017-001) and fed in the laboratory animal center of Anhui University of Chinese Medicine. All rats were housed under the condition of a temperature (22 ± 1°C) and light-controlled (12-hour light-dark cycle) quiet room with humidity of 50% for 7 days to acclimate to the environment before the experiment. The animals had free access to food or water at all time. The experimental operation procedures were in line with the “Guidance Suggestions for the Care and Use of Laboratory Animals” formulated by the Ministry of Science and Technology of China.

### 2.3. Preparation of NLXTD Extract

NLXTD consists of seven medicinal compositions as shown in Table 1. All the herbal constituents were obtained from Guangyintang Chinese Medicine Co., Ltd. (Anhui, China) and carefully authenticated by Associate Professor YU (Anhui University of Traditional Chinese Medicine, Hefei, China) according to the Chinese Pharmacopoeia (2015 edition).

The aqueous extract of NLXTD was prepared by the following processes: Firstly, the components were mixed at the mass ratio of 15 : 5 : 3 : 5 : 2 : 5 : 5 (*Radix Astragali*, *Rhizoma Chuanxiong*, *Radix et Rhizoma Notoginseng*, *Rhizoma Gastrodiae*, *Scolopendra*, *Radix Angelicae Sinensis*, and *Flos Carthami*) and were macerated in 8 volumes of distilled water for 0.5 h. The mixture was boiled for 1 h and the liquids were filtered. Six other volumes of distilled water were added to the residues, decocted, and filtered. Then the powdered medicine of *Scolopendra* was added to the mixed filtered liquids and stirred at remaining temperature until it completely mixed. The final decocted liquids were then centrifuged at low speed (3500 rad/min, 14°C, 10 min) and concentrated by rotary evaporator to 1 mL containing 1.08 g of raw material (equivalent to dry weight). All the decoction was stored at 4°C for use.

### 2.4. Model of Focal Cerebral Ischemia/Reperfusion

In our experiment, the focal cerebral I/R model was established by the right middle cerebral artery occlusion (MCAO) method, which has important applications in many experiments [36, 37]. Briefly, rats were anesthetized by intraperitoneal injection of 3% Pentobarbital sodium and placed on a heating pad at 37°C during the surgery. The right common carotid artery (CCA), external carotid artery (ECA), and the internal carotid artery (ICA) of rats were carefully isolated through a midline incision of the neck about 2 cm. After ligation of the superior thyroid artery and the occipital artery, the distal end of the ECA was ligated and severed. Arterial clamps were placed distal to the right CCA and ICA. Then a 4-0 nylon monofilament (Beijing Cinontech Co., Ltd., China) was inserted into ECA with a distance of 18–20 mm via a small incision and advanced into the ICA in order to obstruct the blood flow into the middle cerebral artery (MCA). After 2 h of occlusion, the nylon line was withdrawn slowly about 5 mm to allow reperfusion of the middle cerebral artery. In the sham group, rats underwent the same operations but without the nylon suture occlusion and reperfusion process. Rats waking up from anesthesia were returned to their cages for free eating and drinking.

### 2.5. Groups and Drug Intervention

Rats were equally and randomly divided into five groups: animals just exposed to the MCA without occlusion and treated with an equal volume of DMSO group (Sham group), treatment of MCAO rats with equal amount of DMSO group (I/R group), MCAO rats treated with the HIF-1*α* inhibitor 2-methoxyestradiol (2ME2, 16 mg/kg) intraperitoneally group (2ME2 group), MCAO rats treated with 2ME2 (16 mg/kg) intraperitoneally, NLXT decoction group (2ME2 + NLXTD group), and MCAO rats treated with NLXT decoction and an equal volume of DMSO group (NLXTD group).

2ME2 is a naturally occurring metabolite of estradiol which is known to depolymerize microtubules and blocks HIF-1*α* transcriptional activity [38]. 2ME2 was purchased from Sigma (St. Louis, MO, USA). The compound of 2ME2 was dissolved in 1% DMSO and further diluted in phosphate buffer saline (PBS) to 2 mL (final <0.01% DMSO). Administration of 2ME2 at the dose of 16 mg/kg 30 minutes before ischemia in rats was based on the optimum dosage performed on the rats [38]. The same volume of DMSO without inhibitor 2ME2 was injected intraperitoneally into rats at the same time. NLXTD at a dose of 8.54 g/kg was administered intragastrically to the rats in the NLXTD group and 2ME2 + NLXTD group according to the body surface area normalization method and human normal clinical usage of crude drug [39]. Intragastrical administration of NLXTD was performed twice a day beginning at 6 h after the operation until sacrificing the rats.

### 2.6. BrdU Labeling

To determine the location and number of proliferating cells, the 5-Bromo-2′-deoxyuridine (BrdU, Solarbio), which is incorporated into S-phase cells during the cell cycle, was consecutively employed. Briefly, starting from ischemia, BrdU (10 mg/mL in saline, i.p.) was injected every four hours for three times to a final dose of 50 mg/kg body weight and for 6 consecutive days [40]. These rats except for TTC staining were sacrificed 24 h after the last injection on the 6th day.

### 2.7. Neurological Deficit Scores

Animal neurological deficit score was evaluated at 1, 3, and 7 days after reperfusion using modified Longa scores [36, 41]: 0, no deficits; 1, difficulty in fully extending the contralateral forelimb; 2, unable to extend the contralateral forelimb; 3, mild circling to the contralateral side; 4, severe circling; and 5, falling to the contralateral side. All rats were evaluated by an independent researcher who was blind to the experiment grouping. Rats with a score of 0 or 5 were excluded from the study.

### 2.8. Measurement of Cerebral Infarct Volumes

Cerebral infarct volumes were measured by TTC staining. 24 hours after MCAO, rats (*n* = 3 per group) were sacrificed by rapid decapitation under deep anesthesia. The whole brain after removal of the olfactory bulb and cerebellum was quickly removed and stored at −20°C freezer for 20 min. The frozen rat brain was sectioned into 5 coronal slices of 2 mm thickness and immersed in a 2% 2,3,5-triphenyltetrazolium chloride (TTC) (Sigma, USA) in the dark for 30 min at 37°C. The infarct tissue showed a white color, while the normal tissue showed a red color. Next, all slices were fixed in 4% paraformaldehyde (Sigma, USA) for 24 h. Finally, brain sections were photographed and analyzed using the ImageJ software (Rawak Software Inc., Germany). The infarct volume was calculated as described by Lin [42].

### 2.9. Hematoxylin & Eosin (H&E) Staining

Seven days after reperfusion, the rats were deeply anesthetized and perfused transcardially with 400 mL 0.9% physiological saline and followed by 300 mL 4% paraformaldehyde in 0.1 M phosphate buffer (pH 7.4). Then, brains were removed and embedded in paraffin routinely and sectioned into slices of 4 *μ*m thickness. Finally, the sections were subjected to standard histochemical staining with hematoxylin and eosin. The morphology was examined under a light microscope (Olympus, Japan) to assess the brain histological injury in the right ischemic penumbra area. Paraffin sections were also used for further experiments.

### 2.10. Terminal Deoxynucleotidyl Transferase dUTP Nick End Labeling (TUNEL) Assay

To detect the possible DNA fragmentation in the cerebral ischemic penumbra area, the TUNEL assay was performed by using an in situ Cell Death Detection Kit (Roche Diagnostics, Mannheim, Germany) in line with the manufacturer's instruction. All brain tissue slices from each group were under the standard histochemical procedure. The quantitative analysis of positively stained nuclei was counted in 3 nonoverlapping microscopic eyeshot and by at least three blind researchers. The apoptosis index (AI) = (the number of TUNEL-positive cells/the total number of cells) × 100%.

### 2.11. Immunohistochemistry (IHC) Staining

For immunohistochemistry staining, the rat brain sections from each group were deparaffinized, rehydrated, and stained using the DAB method under the instruction of the manufacturer. Sections were incubated with rabbit polyclonal against VWF (1 : 200, Zhongshan Biotech. Co., China) overnight at 4°C and then labeled with streptavidin-peroxidase (SP). The test was performed according to the kit instructions. MVD was detected by immunohistochemical staining, and the Weidner method [43] was used for counting. VWF-positive vascular structures or VWF-positive cell populations with or without tube-like structures were classified as microvessels. In the low-power field of view (×40), the most densely distributed VWF-positive blood vessels around the right infarction area were selected; in the high-power field of view (×400), three blood vessel density areas were selected, respectively, and the number of VWF-positive blood vessels was counted. The average value was the average number of microvessels of the sample.

### 2.12. Immunofluorescence (IF) Staining

Double-label immunofluorescent staining for lectin was performed to detect angiogenesis around the penumbra region on day 7 after occlusion. Rats were anesthetized at 7 days after the operation, and the brains were transcardially perfused with NS followed by 4% paraformaldehyde. After gradient elution with sucrose, the brains were quickly frozen and cut into 6 µm coronally thick sections. These sections were permeabilized with 0.5% Triton X-100 for 5 min and blocked with 10% donkey serum for 1 h. Then, sections were incubated with anti-BrdU (1 : 500, Abcam NBP2-29414) and anti-CD34 (1 : 100, Santa Cruz Biotechnology, USA) overnight at 4°C. Next, the sections were briefly washed with PBST and incubated with donkey anti-mouse secondary antibody (1 : 400, Abcam, United States) for 1 h at 37°C. After counterstaining with 4,6-diamidino-2-phenylindole (DAPI; Solarbio, Beijing, China), the double-stained sections on the right side were observed and photographed using a fluorescence microscope, and analysis was conducted with the ImageJ software (Rawak Software Inc., Germany).

### 2.13. Western Blotting Analysis

Western immunoblot analysis was used to assess protein expression of HIF-1*α*, VEGF, VEGFR2, and Notch1. Three rats of each group were sacrificed after anesthesia. Right brains tissues were removed immediately and stored at −80°C until analysis. The proteins were isolated from the ischemic cortex tissue after lysis in RIPA buffer (Beyotime, Shanghai, China), and protein determination was quantitated using a BCA Protein Assay kit (Beyotime, Shanghai, China). Denatured protein samples were separated by sodium dodecyl sulfate-polyacrylamide gels (SDS-PAGE) and transferred to polyvinylidene fluoride (PVDF) membranes. After blocking with 5% nonfat milk at room temperature for 1 h, the membranes were incubated with primary antibodies: rabbit polyclonal anti-HIF-1*α* (1 : 500, BIOSS) and rabbit polyclonal antibody anti-VEGF (1 : 500, BIOSS), overnight at 4°C. Then, after rinsing in TBST buffer for 5 min three times, the membrane was incubated with an anti-rabbit or anti-mouse horseradish peroxidase-conjugated secondary antibody (1 : 10000, Santa Cruz Biotechnology, Santa Cruz, CA) at room temperature for 2 h. Finally, the protein bands were visualized using enhanced chemiluminescence (ECL) detection kit (Yeasen Biotech, Shanghai, China) and were quantitatively analyzed by using ImageJ software (Rawak Software Inc., Germany).

### 2.14. Quantitative RT-PCR Analysis

The total RNA was extracted in strict accordance with the instructions of the TRIzol kit reagent (Invitrogen, United States). RNA samples were purified with a genomic DNA eliminator by using the RNeasy Micro Kit (Qiagen, Valencia, CA, United States) and reverse-transcribed into cDNA by using PrimeScript RT Reagent Kit (Invitrogen, United States).

Quantitative RT-qPCR procedure was performed on a LightCycler thermal cycler system (Bio-Rad, United States) using a fluorescent dye (SYBR Green I, Takara) and gene-specific primers according to the following protocol: denaturation at 94°C for 3 min, 40 cycles of 94°C for 5 sec and 34 sec at 60°C. The sequences of gene-specific primers were as shown in Table 2.

### 2.15. Statistical Analysis

All statistical analyses were conducted with SPSS software (version 20.0). The data were expressed as means ± standard deviation (mean ± SD). Differences between more than two groups were analyzed using one-way analysis of variance (ANOVA) followed by Tukey's post hoc analysis, and Student's *t*-test was used for two-group comparison. *P* < 0.05 for the difference between groups was considered statistically significant, and EXCEL 2010 software was used to draw the statistical chart.

## 3. Results

### 3.1. Neuroprotective Effects of NLXTD following Cerebral I/R Injury

**Figure 1:**
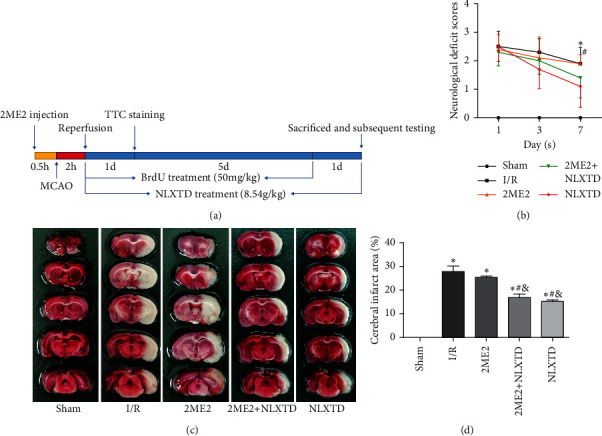
Effect of NLXTD on neurological deficit score and cerebral infarct volume in rats after cerebral I/R. (a) The schematic diagram of the experiment. (b) The neurological deficit scores in each group after MCAO at 1, 3, and 7 days. Values are expressed as the mean ± SD (*n* = 10); ^*∗*^*P* < 0.05 versus the I/R group; ^#^*P* < 0.05 versus the NLXTD group. (c) Representative images of TTC staining sections in rats of different groups at 24 hours after MCAO. (d) The quantitative analysis of infarct volumes. The data are presented as the mean ± SD (*n* = 3). ^*∗*^*P* < 0.05 versus the sham group; ^#^*P* < 0.05 versus the I/R group; ^&^*P* < 0.05 versus the 2ME2 group.

Except for the TTC-stained rats, we continuously detected the neurological deficit scores of 10 rats in each group at 1, 3, and 7 days after MCAO as shown in Figure 1(b). No neurological deficits were observed in the sham group. Compared with the I/R group, the neurological deficit scores were significantly decreased in NLXTD-treated rats at 7 days after MCAO (neurological deficit score: 1.1 ± 0.738 in the NLXTD group versus 1.9 ± 0.568 in the I/R group, *P* < 0.05), while the HIF-1*α* inhibitor 2-methoxyestradiol (2ME2) treatment increased the neurological deficit scores at 7 days compared with the 2ME2 + NLXTD group but without statistically significant difference. After cerebral I/R injury, the NLXTD group had lower neurological deficit scores at 7 days compared with the 2ME2 group with the significantly statistical difference (neurological deficit score: 1.1 ± 0.738 in the NLXTD group versus 1.9 ± 0.316 in the 2ME2 group, *P* < 0.05).

In the sham-operation group, red staining was uniform, and no infarcts were observed. Compared with the sham group, the rats in the I/R group had obvious infarcts in brain tissue. Moreover, the infarct volume was markedly reduced in the NLXTD-treated groups (2ME2 + NLXTD and NLXTD group) compared with that in the I/R group (*P* < 0.05). However, the effects of NLXTD on infarct volumes were attenuated by the 2ME2 inhibitor (25.36 ± 0.6 in the 2ME2 group versus 16.82 ± 1.46 in the 2ME2 + NLXTD group, *P* < 0.05) (Figures 1(c) and 1(d)).

These findings indicated that NLXTD had a neuroprotective effect on neurons, while 2ME2 attenuated the protective effect of NLXTD against cerebral I/R.

### 3.2. Effect of NLXTD on Pathological Changes and Neuron Apoptosis on Cerebral I/R Injury

**Figure 2:**
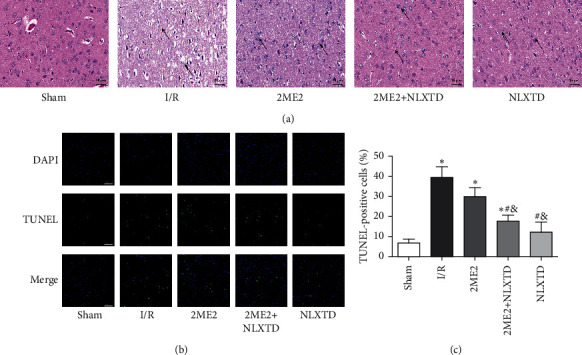
H&E staining and TUNEL staining in the ischemic penumbra on the 7th day after MCAO. (a) Histopathological examination results of neurons in cerebral peri-infarct cortex of rats in each group (*n* = 3). The black arrows indicate typical changes. (b) TUNEL staining showed neuronal apoptosis in all groups. Nuclei were stained with DAPI (blue), and the colocalization of blue and green indicates TUNEL-positive cells. Scale bars = 40 *μ*m; magnification: 400×. (c) Quantitative analysis of neuronal apoptosis. Data are presented as the mean ± SD (*n* = 4); ^*∗*^*P* < 0.05 versus the sham group; ^#^*P* < 0.05 versus the I/R group; ^&^*P* < 0.05 versus the 2ME2 group.

The H&E staining and TUNEL staining assay were employed to evaluate the pathological changes and neuron apoptosis in the rat ischemic border zone. The cortical cells in the sham-operated group were neatly lined up. In the I/R group, there were typical ischemic changes: disordered structure, obvious edema, vacuolization, and nuclear pyknosis. Compared with the I/R group, those in the NLXTD-treated groups showed fewer brain pathological changes because the internal cell structure was more complete, the edema was reduced, and the number of surviving neurons increased. In contrast with the 2ME2 + NLXTD group, more pathological changes in the MCAO with the 2ME2 group were observed (Figure 2(a)).

The TUNEL assay was also used to detect apoptosis during this process. As shown in Figure 2(b), there are few TUNEL-positive cells in the sham and NLXTD groups. The apoptotic neuronal cells were expressed most in the I/R group. Compared with the I/R group, the numbers of TUNEL-positive cells of 2ME2 + NLXTD and NLXTD groups were significantly decreased (*P* < 0.05). Similarly, although the number of TUNEL-positive cells in the 2ME2 group was lower than that in the I/R group, it was significantly higher than that of the 2ME2 + NLXTD group (*P* < 0.05, Figure 2(c)). These results indicated that 2ME2 inhibited the neuroprotective effect of NLXTD on cerebral I/R injury.

### 3.3. Effect of NLXTD on Microvessel Density (MVD) on Cerebral I/R Injury Rats

**Figure 3:**
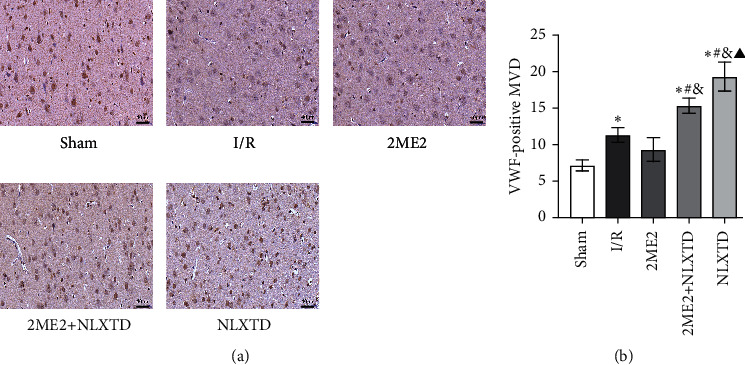
Effects of NLXTD on microvascular density around the infarction in cortex. (a) The VWF expression in the cerebral cortex infarction by immunohistochemical staining. (b) Quantitative analysis of microvascular density (mean ± SD, *n* = 6). Scale bar: 40 *μ*m; magnification: 400×. ^*∗*^*P* < 0.05 versus the sham group; ^#^*P* < 0.05 versus the I/R group; ^&^*P* < 0.05 versus the 2ME2 group; ^▲^*P* < 0.05 versus the 2ME2 + NLXTD group.

VWF-positive vessels were detected visibly in the cortex around the cerebral penumbra in groups after 7 days of MCAO/R. In the I/R group, MVD significantly increased at 7 days compared with the sham group (*P* < 0.05). Similarly, MVD in the NLXTD group significantly increased at 7 days compared with the I/R group (*P* < 0.05). However, the inhibitor 2ME2 attenuated the NLXTD's protective effect on promoting the number of microvascular densities. Compared with the NLXTD group, MVD significantly decreased in the 2ME2 + NLXTD group at 7 days (*P* < 0.05). MVD decreased in the 2ME2 group when compared with the I/R group at 7 days without a significant difference. MVD had a significant difference between the 2ME2 + NLXTD group and the 2ME2 group at 7 days (*P* < 0.05). Moreover, the microvascular density in the brain penumbra tissue was the highest in the MCAO/R rats treated with NLXTD. These results indicated that 2ME2 inhibited the revascularization effect of NLXTD on cerebral I/R injury (Figures 3(a) and 3(b)).

### 3.4. Effect of NLXTD on Angiogenesis (Neovascularized Endothelial Cells) in the Penumbra of Rats following Cerebral I/R Injury

**Figure 4:**
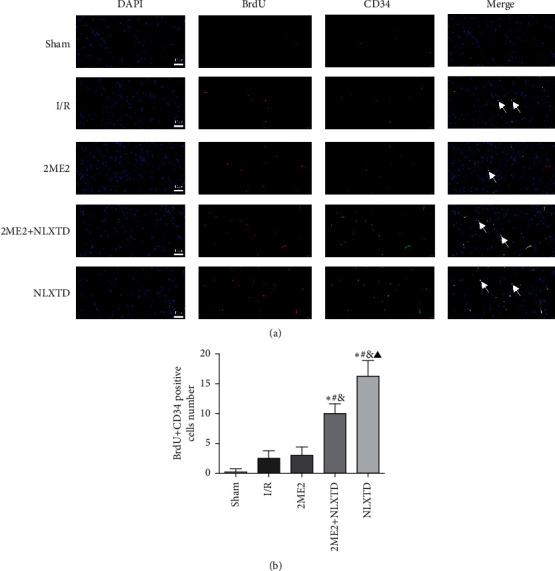
Immunofluorescence staining of neovascularization with BrdU + CD34 double-stained around the penumbra area. (a) The colocalization cells presented. The white arrows indicate the double-stained cells; (b) BrdU + CD34 positive cells count in each group. Scale bars = 40 *μ*m; magnification: 400×. Data are expressed as the mean ± SD (*n* = 4); ^*∗*^*P* < 0.05 versus the sham group; ^#^*P* < 0.05 versus the I/R group; ^&^*P* < 0.05 versus the 2ME2 group; ^▲^*P* < 0.05 versus the 2ME2 + NLXTD group.

BrdU is commonly used to determine the location and number of proliferating cells. CD34 molecule is a member of the cadherin family and it is used as a marker for vascular endothelium [44]. Therefore, the formation of vascular endothelial cells in ischemic regions was characterized by immunofluorescence double-labeling staining. The experiment results showed that the BrdU + CD34 positive colabeling cell numbers in the area around the ischemic lesion were significantly higher in the NLXTD group at 7 days compared with the I/R group (*P* < 0.05) (Figure 4(b)). Although the number of neovascularized endothelial cells in the 2ME2 + NLXTD group was also increased compared to the sham and 2ME2 groups, its increase was significantly lowered after NLXTD treatment (10 ± 1.63 in the 2ME2 + NLXTD group versus 16.25 ± 2.63 in the NLXTD group, *P* < 0.05). These results demonstrated that the treatment of NLXTD promoted angiogenesis and improved the integrity of vascular structures in the infarcted zone, while the inhibitor 2ME2 played the opposite effect.

### 3.5. NLXTD Mediated the Expression of Proteins in HIF-1*α*/VEGF Signaling Pathway

In order to investigate the potential mechanism of the NLXTD-activated angiogenesis protective effects on cerebral I/R injury, we evaluated the expression of proteins in the HIF-1*α*/VEGF signaling pathway and Notch1 by western blotting assay. The levels of HIF-1*α*, VEGF, and Notch1 mRNA in the ischemic penumbra were examined by quantitative RT-PCR analysis in all groups.

**Figure 5:**
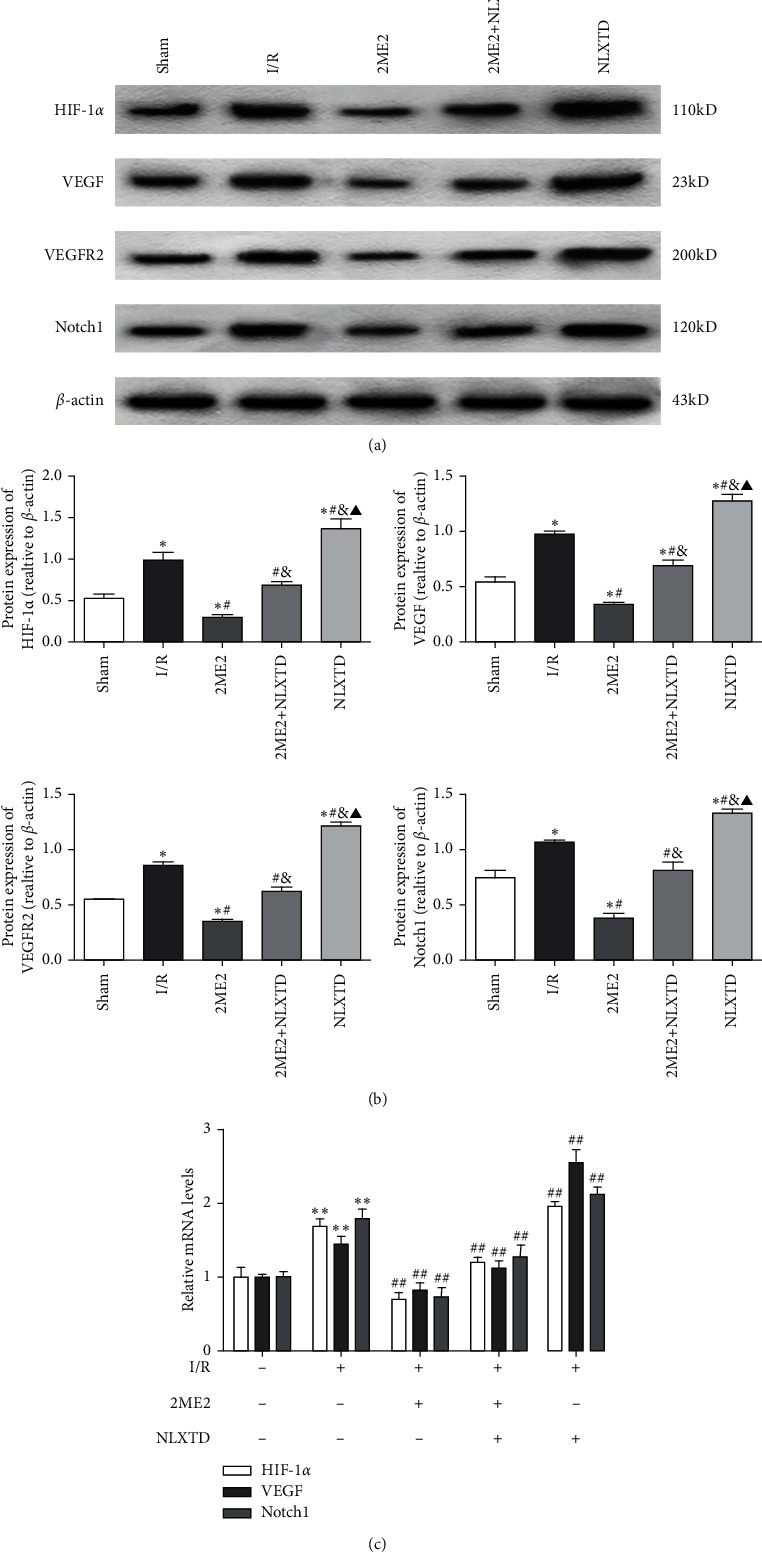
NLXTD induced angiogenesis factor expression and activated the HIF-1*α*/VEGF signaling pathway at 7 d after reperfusion. (a) Representing western blot analysis of HIF-1*α*, VEGF, VEGFR2, and Notch1. (b) Quantitative analysis of protein levels of HIF-1*α*, VEGF, VEGFR2, and Notch1. *β*-Actin was used as a loading control. Data are expressed as the mean ± SD(*n* = 3); ^*∗*^*P* < 0.05 versus the sham group; ^#^*P* < 0.05 versus the I/R group; ^&^*P* < 0.05 versus the 2ME2 group; ^▲^*P* < 0.05 versus the 2ME2 + NLXTD group. (c) The mRNA expressions of HIF-1*α*, VEGF, and Notch1 (mean ± SD, *n* = 3). ^*∗*^*P* < 0.05 and ^∗∗^*P* < 0.01 versus the sham group; ^#^*P* < 0.05 and ^##^*P* < 0.01 versus the I/R group.

The western blotting results demonstrated that the expression levels of HIF-1*α*, VEGF, and VEGFR2 protein in the I/R group were markedly enhanced, and NLXTD application obviously increased the expression compared with the I/R group (Figure 5(b), *P* < 0.05). In addition, NLXTD could dramatically upregulate the Notch1 protein expression. However, the 2ME2 treatment significantly attenuated HIF-1*α* protein expression when compared with the I/R group (*P* < 0.05). Interestingly, the HIF-1*α* inhibitor 2ME2 can inhibit the increase of both VEGF and VEGFR2 expression caused by 2ME2 with NLXTD treatment, which suggested that HIF-1*α*/VEGF signaling was probably involved in rats induced by brain injury. Moreover, a similar reduced effect was observed in Notch1 protein expression in the 2ME2 + NLXTD group compared with the NLXTD group.

Finally, we determined the mRNA expression levels of HIF-1*α*, VEGF, and Notch1 in the injured brain tissues by quantitative RT-PCR analysis. Quantitative RT-PCR results showed that the mRNA expression levels of HIF-1*α*, VEGF, and Notch1 in the I/R group were higher than those of the sham group (*P* < 0.01, Figure 5(c)). As expected, mRNA expressions of HIF-1*α*, VEGF, and Notch1 in the NLXTD groups were significantly upregulated compared with the I/R group (*P* < 0.01). Consistent with the western blotting data results, all the detected mRNAs expressions were significantly downregulated by treatment of 2ME2 (*P* < 0.01).

In conclusion, western blot and quantitative RT-PCR analysis showed similar expression trends of HIF-1*α*, VEGF, VEGFR2, and the Notch1 proteins or mRNAs in the right brain ischemic penumbra. After I/R injury, protein or mRNA expression was increased and further promoted by NLXTD, while the treatment of 2ME2 reduced the increase of this expression. Furthermore, our results showed that the expression level of proteins in the 2ME2 group was the lowest among all the groups. Finally, we demonstrated that NLXTD can obviously activate the HIF-1*α*/VEGF signaling pathway.

## 4. Discussion

Reperfusion injury is a complex pathological process involving multiple cytokines and signaling pathways, and its neurological damage signs and morphological changes are sometimes more obvious than the previous results [45]. After cerebral ischemia, blood flow in the brain penumbra area is reduced but not completely blocked. It has been reported that the angiogenesis phenomenon is more common for 1 to 2 weeks in MCAO/R rats and especially obvious in the ischemic penumbra zone [46]. The present studies indicate that angiogenesis plays a key role in the recovery of neurological function after stroke because angiogenesis leads to the formation of new blood vessels and increasing the oxygen, glucose, nutrients, and neural stem cells (NSCs) migration to the ischemic region [47, 48]. However, the self-repair ability around the ischemic tissue is limited, which cannot improve the blood supply of neurons and nerve function recovery after cerebral ischemia. Thus, it is very important to explore effective methods to restore blood supply and enhance angiogenesis in the ischemic area as soon as possible, which is conducive to the recovery of nerve function timely.

In recent years, TCM has gradually become a promising treatment approach for cerebral ischemia disease because it has the characteristics of multiple targets and multiple components [49, 50]. As a well-known traditional Chinese herbal formula, NLXTD, involving six plant medicines and one animal medicine, has been widely used in clinical treatment of ischemic stroke [22]. A recent study has shown that NLXTD contains some effective chemical constituents, including but not limited to the following: Calycosin, Calycosin-7-O-ꞵ-D-glucoside, and Ononin from Astragali Radix, Ligustrazine from Chuanxiong Rhizoma, 4-hydroxbenzyl alcohol and Parishin A from Gastrodiae rhizome, and ferulic acid from Angelicae Sinensis Radix [19]. According to our previous research, NLXTD on cerebral I/R injury mainly focused on its effects of inhibiting inflammatory response, inhibiting apoptosis, and promoting nerve regeneration [20, 21, 51]. However, the role of NLXTD in promoting angiogenesis and neuroprotection remains unclear. Therefore, this study provides evidence for one of the molecular mechanisms about how NLXTD promotes angiogenesis and neuroprotection in MCAO rats.

Hypoxia-inducible factor-1*α* (HIF-1*α*), an important upstream transcriptional factor induced by low oxygen, modulates the expression of multiple genes responding to various pathophysiological conditions associated with hypoxia [52]. When tissues or cells are in an anoxic environment, HIF-1*α* expression is increased, which may specifically bind the anoxic response elements of the vascular endothelial growth factor (VEGF) promoter, enhancing the stability of the biological function of VEGF and promoting the expression of VEGF [53]. Accumulating evidence has shown that the primary function of VEGF in ischemic disease is associated with neuroprotective effect by reducing cell death and promoting angiogenesis [54]. In addition, previous studies using a focal or global cerebral ischemia model have found that overexpression of HIF-1*α* was involved in various adaptive responses and protected the neuronal cells from ischemia- or hypoxia-induced damages in vitro or in vivo [27, 55]. However, at present, research on the role of HIF-1*α*/VEGF signaling pathways serving in the response to cerebral I/R injury is very limited and unclear.

In our study, a rat cerebral I/R model was established to confirm that NLXTD had the effects of reducing cerebral ischemic neurological damage, cerebral infarction size, reducing the number of damaged apoptotic cells, and increasing the MVD and the number of cerebral neovascularized endothelial cells in the boundary ischemic area. These results suggest that NLXTD has neuroprotective effect on cerebral ischemia. Further investigation of the mechanism indicated that the promotion of angiogenesis after NLXTD therapy was regulated by HIF-1*α*/VEGF signaling pathway.

Angiogenesis is essential for the recovery of neurological functional after stroke [48]. The formation of blood vessels allows the blood flow in the ischemic penumbra and protects the ischemic brain from injury. The clinical study verified that increasing angiogenesis is an effective method to improve the prognosis of stroke patients [14]. To further observe the effect of NLXTD on angiogenesis, we selected a series of protein indicators for research. CD34 is a marker of vascular endothelial cells, which can not only stain mature preexisting vessels but also stain newly formed blood vessels [56]. It can be used to evaluate the density of microvessels and it is an indirect marker of angiogenesis [57]. In our study, the newborn vascular endothelial cells double-labeled with BrdU and CD34 were observed around the penumbra area following cerebral I/R injury, along with a significantly increased quantity after the NLXTD treatment, directly verifying the angiogenesis effects of NLXTD. In contrast, inhibition of HIF-1*α* significantly decreased double-labeled cells number, indicating the angiogenesis effects of HIF-1*α* on I/R rats. In addition, the present experimental results also showed that the microvascular density was the highest in the MCAO/R rats treated with NLXTD (*P* < 0.05,Figure 3). Neovascularization secretes neurotrophic factors and chemokines, which may create a suitable microenvironment in the damaged brain to support the survival of newly formed neurons [58]. In our study, the H&E staining, TUNEL assay, and microvessel density were used to evaluate the angiogenesis. Here, we were pleased to find that the number of neuronal apoptotic cells decreased and the neurological deficit score decreased after NLXTD treatment. However, recovery of neurological function is not entirely dependent on promotion and coordination of angiogenesis of ischemic tissue [59]. Other pathophysiological and biochemical reactions in ischemic tissue, such as free radical release, inflammatory response, and cell death, can also interfere with the recovery of neuronal cell function [60]. Our results suggest that angiogenesis and neurological recovery are consistent.

It has been reported that induction of endogenous VEGF has a direct neuroprotective effect on cerebral ischemia [61, 62], and the neuroprotective mechanism of VEGF may be related to angiogenesis [63] or vascular protection. In addition, VEGF has neurotrophic and neuroprotective activities, and its direct neurotrophic effect may provide neuroprotection independent of angiogenesis [64]. As one of the high-affinity receptors for specific binding to VEGF, VEGFR2 is involved in all aspects of normal and pathologic vascular endothelial cell biology. VEGFR2 also plays a vital role in vascular remodeling and neural protection after 7 days of reperfusion [65–67]. Our research showed that ischemia-induced VEGF and VEGFR2 expression was upregulated by NLXTD treatment, together with upregulation of HIF-1*α* and Notch1 proteins. In contrast, inhibition of HIF-1*α* by 2ME2 abolished the protein level of VEGF and VEGFR2 mediated by NLXTD, suggesting that NLXTD promoted angiogenesis in cerebral I/R injury via upregulating the expression of HIF-1*α*.

In recent years, mounting evidence has proved that the Notch signaling pathway is involved in the process of angiogenesis, which may be related to the modulation of VEGF [68, 69]. It has been reported that the Notch signaling pathway is highly interacted with the VEGF pathway: VEGF induces DLL4/Notch signaling at several levels, while Notch signaling regulates the VEGF pathway, leading to the formation of embryonic vascular development and tumor angiogenesis [70]. Using a focal transient ischemic stroke model, Wang [71] found that not only HIF-1*α*/VEGF but also DLL4/Notch is involved in a dl-NBP-induced increase of the number of blood vessels and promoting the functional recovery. Meanwhile, an in vivo study of Qi [72] showed that the overexpression of Notch1 or Jagged1 in the Notch signaling pathway enhances the migration, invasiveness, and angiogenic ability of endothelial progenitor cells in a rat model of traumatic brain injury. However, inhibition of the Notch signaling pathway with Notch1 or Jagged1 siRNAs reduced the ability of blood vessel formation and tissue repair. Consistent with the above findings, the expression of Notch1 signifiantly decreased at 7 days in the I/R group. NLXTD could further upregulate the expression of Notch1 after 7 days of reperfusion. Interestingly, there were similar expression trends between Notch1, HIF-1*α*, and VEGF. In addition, the Notch signaling pathway also plays a crucial role in various biological events, including the maintenance of stem cells, the specification of cell fate, cell survival, and the formation of tissue morphology [73–75]. Liquan Wu et al. reported that overexpression of circCCDC9 protected the blood-brain barrier and inhibited apoptosis by suppressing the Notch1 signaling pathway [76]. In our study, results of TUNEL staining showed that NLXTD treatment could significantly reduce the number of apoptotic neurons, which may be related to the activation of Notch1 signaling pathway. In the meantime, the administration of 2ME2 dramatically suppressed the induction of Notch1 and increased the number of apoptotic cells, indirectly verifying that the increasing expression of Notch1 in I/R injury is related to the HIF-1*α*/VEGF signaling pathway and NLXTD treatment had a positive effect on I/R rats.

Conflicting reports have aroused many debates on the certain role of HIF-1*α* in rats after cerebral ischemia. Some studies clearly showed that HIF-1*α* exerts a neuroprotection role by correlating with the expression of target genes involved in various adaptive responses in cerebral ischemia [25, 55], while other experimental studies demonstrated that HIF-1*α* played a harmful effect by activating the expression of multiple prodeath genes after stroke, and the HIF-1*α* inhibition was beneficial [77, 78]. Further study found that HIF-1*α* activation had two phases after MCAO [27]. The initial phase occurred after occlusion injury till 24 h and was associated with upregulating various HIF-1 target genes, including most of the genes that promote death. However, in the second phase, the activation of HIF-1*α* lasted up to 10 d, and prodeath genes such as BNIP3 and Noxa remained not elevated, whereas the expression of target genes involved in angiogenesis continued to be higher in the ischemic hemisphere. It may indicate that time distinguished the different effects of HIF-1*α* in cerebral ischemia. Although the previous evidence is paradoxical, our data clearly showed that NLXTD had a neuroprotective and angiogenesis function against I/R injury in rats, together with the motivation of the HIF-1*α*/VEGF signaling pathway. The decline of neuronal apoptosis and microvascular density caused by MCAO can be blocked by NLXTD in the ischemic penumbra.

Nevertheless, there were still several limitations in this paper. Our current study focused more on the effect of traditional Chinese medical recipe NLXTD treatment on the promotion of angiogenesis than on the inhibition of neuronal apoptosis and other mechanisms, which is a limitation of our research. Our future work will pay more attention to effects of NLXTD treatment for ischemic stroke and other regulatory mechanisms. Another limitation of our research is that we only focused on the effect of NLXTD on targeting the HIF-1*α*/VEGF signaling pathway in promoting angiogenesis after 7 days of a stroke, which lacked a dynamic observation of indicators. Recent research has shown that NLXTD treatment has a therapeutic effect on IS from day 1 to day 7 [51]. Our future work will focus more on the effects of NLXTD treatment on MCAO rats at different time points and expand the sample size accordingly.

Our research provides a potential experimental basis for the clinical application of TCM in the treatment of ischemic stroke. Therefore, further studies are required to investigate the detailed mechanism. Furthermore, NLXTD prescription is a complex multicomponent and multitarget system, so the specific constituents of NLXTD responsible for the neuroprotective and angiogenesis effects need to be further explored.

## 5. Conclusion

In summary, this study is the first to demonstrate that NLXTD can alleviate cerebral I/R injury by enhancing angiogenesis, which is associated with the HIF-1*α*/VEGF signaling pathway. Our findings provided a novel insight into the role of NLXTD against ischemic stroke and suggested that NLXTD may be an effective therapeutic agent for stroke therapy. These findings also provide the following implications for future research: (1) the relationship between angiogenesis and neuronal apoptosis, (2) the interaction between HIF-1*α*/VEGF and DLL4/Notch signaling pathway on the angiogenesis, and (3) comprehensive effect of NLXTD in the treatment of ischemic stroke.

## Figures and Tables

**Table 1:** The composition of NLXTD.

Latin name	Chinese herbal name	Medicinal parts	Amount used (g)	Weight ratio (%)
Radix Astragali	Huangqi	Roots	30	37.5
Rhizoma Chuanxiong	Chuanxiong	Rhizomas	10	12.5
Radix et Rhizoma Notoginseng	Sanqi	Radix et Rhizome	6	7.5
Rhizoma Gastrodiae	Tianma	Rhizomas	10	12.5
*Scolopendra*	Wugong	All	4 (two slices)	5.0
Radix Angelicae Sinensis	Danggui	Roots	10	12.5
Flos Carthami	Honghua	Flowers	10	12.5
Total amount			80	100

**Table 2:** Sequences of primers for quantitative RT-PCR.

Gene	Primers	Sequences	Amplicon size (bp)
*β-Actin*	Forward	5′-CCCATCTATGAGGGTTACGC-3′	150
	Reverse	5′-TTTAATGTCACGCACGATTTC-3′	

HIF-1*α*	Forward	5′-TGACCACTGCTAAGGCATCA-3′	111
	Reverse	5′-GGCTCCTTGGATGAGCTTTG-3′	

*VEGF*	Forward	5′-TTGAGACCCTGGTGGACATC-3′	116
	Reverse	5′-CTCCAGGGCTTCATCATTGC-3′	

Notch1	Forward	5′-GTGTGAGTCCAACCCTTGTG-3′	176
	Reverse	5′-ATTTGTACCCAGCGACATCA-3′	

## Data Availability

No underlying data were used in this study.

## Abbreviations

BrdU: 5-Bromo-2′-deoxyuridine
H&E: Hematoxylin & eosin
HIF-1*α*: Hypoxia-inducible factor-1*α*
I/R: Ischemia-reperfusion
MCAO: Middle cerebral artery occlusion
MVD: Microvessel density
NLXTD: Naoluo Xintong decoction
rtPA: Recombinant tissue plasminogen activator
TCM: Traditional Chinese medicine
TTC: 2, 3, 5-Triphenyltetrazolium chloride
TUNEL: Terminal deoxynucleotidyl transferase dUTP Nick end labeling
VEGF: Vascular endothelial growth factor
2ME2: 2-Methoxyestradiol.
